# The Possible Mechanisms of HSV-TK/Hyperthermia Combined with 131I-antiAFPMcAb-GCV Nanospheres to Treat Hepatoma

**DOI:** 10.1155/2018/8941908

**Published:** 2018-05-03

**Authors:** Mei Lin, Chenglin Zhou, Junxing Huang, Weizhong Tian, Hong Yu, Xingmao Jiang, Jun Ye, Ting Guo, Yujuan Shi, Yanhong Xiao, Xuefeng Bian, Xiaoqian Feng

**Affiliations:** ^1^Clinical Laboratory, Taizhou People's Hospital Affiliated to Nantong University, Taizhou, Jiangsu 225300, China; ^2^Oncology Department, Taizhou People's Hospital Affiliated to Nantong University, Taizhou, Jiangsu 225300, China; ^3^Imaging Department, Taizhou People's Hospital Affiliated to Nantong University, Taizhou, Jiangsu 225300, China; ^4^Pathology Department, Taizhou People's Hospital Affiliated to Nantong University, Taizhou, Jiangsu 225300, China; ^5^School of Chemical Engineering and Pharmacy, Wuhan Institute of Technology, Wuhan, Hubei 430000, China; ^6^Institute of Clinical Medicine, Taizhou People's Hospital Affiliated to Nantong University, Taizhou, Jiangsu 225300, China

## Abstract

Our previous findings showed a good therapeutic effect of the combination of suicide gene HSV-TK, nuclide 131I, and magnetic fluid hyperthermia (MFH) on hepatoma by using magnetic nanoparticles as linkers, far better than any monotherapy involved, with no adverse effects. This combination therapy might be an eligible strategy to treat hepatic cancer. However, it is not clear how the combination regimen took the therapeutic effects. In the current study, to explore the possible mechanisms of radionuclide-gene therapy combined with MFH to treat hepatoma at tissue, cellular, and molecular levels and to provide theoretical and experimental data for its clinical application, we examined the apoptosis induction of the combination therapy and investigated the expression of the proteins related to apoptosis such as survivin, livin, bcl-2, p53, and nucleus protein Ki67 involved in cell proliferation, detected VEGF, and MVD involved in angiogenesis of tumor tissues and analyzed the pathologic changes after treatment. The results showed that the combination therapy significantly induced the hepatoma cell apoptosis. The expression of survivin, VEGF, bcl-2, p53, livin, Ki67, and VEGF proteins and microvascular density (MVD) were all decreased after treatment. The therapeutic mechanisms may be involved in the downregulation of Ki67 expression leading to tumor cell proliferation repression and inhibition of survivin, bcl-2, p53, and livin protein expression inducing tumor cell apoptosis, negatively regulating VEGF protein expression, and reducing vascular endothelial cells, which results in tumor angiogenesis inhibition and microvascular density decrease and tumor cell necrosis. These findings offer another basic data support and theoretical foundation for the clinical application of the combination therapy.

## 1. Introduction

As we all know, cancer has become the leading killer that endangers human health, with the highest morbidity and mortality. Undoubtedly, radiotherapy, chemotherapy, thermotherapy, and biotherapy all contribute to antitumor treatment to a great extent, but each has its own advantages and disadvantages, and any of them can hardly cure cancer thoroughly. Inspiring, comprehensive treatment, a joint therapeutic strategy based on multidiscipline and (or) multimethod by a specific way in view of their respective properties, has shown a great potential for malignancy treatment. It is not a simple overlap of some protocols but is put into use rationally to complement each other's advantages, resulting in an effective synergism [1]. Studies have shown that the combination of more than two therapeutic regimens can get better antitumor effects than any of the monotherapies involved [1–5]. In our previous study, we organically combined suicide gene, internal irradiation of nuclide, and magnetic fluid hyperthermia (MFH) to treat hepatoma by employing magnetic nanoparticles as hinges. In detail, pHRE-Egr1-HSV-TK was transfected into hepatoma cells by using PEI-Mn_0.5_Zn_0.5_Fe_2_O_4_ nanoparticles (PEI-MZF-NPs) as the gene transfer vector, and subsequently 131I-antiAFP McAb-GCV-BSA-NPs were intervened into hepatoma, and then the tumors were directionally heated in an alternating magnetic field by adopting PEI-MZF-NPs as magnetic media. Thus, while 131I and hyperthermia killing tumor cells, nuclide irradiation enabled the Egr1 promotor to induce HSV-TK gene to express, and the expression could be especially enhanced by HRE in hypoxic solid cancer, causing a multiple targeted killing effect of genes, radionuclide and hyperpyrexia against hepatoma. The results demonstrated that the radionuclide-gene combined with MFH had a good therapeutic effect on hepatoma, far better than any of the monotherapies; furthermore, no significant side effects were found [1]. It might be an applicable strategy for hepatic cancer treatment. However, how did the combination therapy exert therapeutic effects on hepatoma? What was the mechanism? This was unclear.

It is understood that radiotherapy, chemotherapy, gene therapy, and thermotherapy have their own antitumor mechanisms, which are comprehensive and complicated. They may play antitumor roles in various ways. For instance, they may induce tumor cell apoptosis, restrain cell proliferation, inhibit tumor angiogenesis, or induce necrocytosis. Their antitumor effects may also be the result of joint action of various ways [6–9]. In the current study, to further investigate the antihepatoma effect of the radionuclide-gene therapy combined with MFH, explore the possible mechanisms at tissue, cellular, and molecular levels, and provide theoretical evidences and experimental data for its clinical application, the apoptotic induction of the combination therapy was examined; the expression changes of the proteins related to apoptosis such as survivin, livin, bcl-2, p53, and nucleus protein Ki67 involved in cell proliferation were analyzed; VEGF and MVD related to angiogenesis in the tumor tissues were detected; and pathologic changes after treatment were observed.

## 2. Materials and Methods

### 2.1. Main Materials

The Annexin V-FITC/PI kit was purchased from Invitrogen Corporation. Survivin, Ki67, livin, bcl-2, p53, VEGF, and MVD immunohistochemistry kits were purchased from Cell Signaling Technology Corporation. HepG2 cell line was provided by the Institute of Biochemistry and Cell Biology, Shanghai Institute of Biological Sciences, Chinese Academy of Sciences. PHRE-Egr1-HSV-TK was constructed previously [1]. Hepatic cancer tissues of the control group, the radionuclide group, the MFH group, the radionuclide-gene group, and the radionuclide-gene-MFH group came from the previous study [1].

### 2.2. Methods

#### 2.2.1. Cell Apoptosis Analyzed by the Flow Cytometer

PHRE-Egr1-HSV-TK was transfected via PEI-MZF-NPs as the reference [1]. The transfected cells (HepG2/TK cells) were subcultivated in a culture plate with six wells. The experiment fell into these groups: (1) the negative control group (HepG2 cells without transfection); (2) the 131I group (HepG2 cells without transfection, known as the nuclide group); (3) the radionuclide-gene group (the pHRE-Egr1-HSV-TK/131I-antiAFPMcAb-GCVgroup); (4) the MFH group (HepG2 cells without transfection); and (5) the radionuclide-gene-MFH group (the pHRE-Egr1-HSV-TK/131I-antiAFPMcAb-GCV/MFH group). After incubation for 24 h, each group was correspondingly added with 131I-antiAFPMcAb-GCV-BSA-NPs (final concentration: 200 *μ*Ci), PEI-MZF-NPs (final concentration: 10 g/l), 131I (final concentration: 200 *μ*Ci), and DMEM. Group (3) was incubated for 48 h at 37°C under the condition of 0.1% O_2_, 5% CO_2_, and N_2_ balance gas (hypoxic conditions). After incubation for 24 h in routine conditions, group (4) was heated for 1 h by a high frequency heater (4 kw, 230 Hz, 30 A) and then continued to be cultured for 23 h in routine conditions. After incubation for 24 h at 37°C under the above hypoxic condition, group (5) was heated for 1 h by a high-frequency heater (4 kW, 230 Hz, 30 A) and then continued to be cultured for 23 h at 37°C under hypoxic conditions. Group (1) and (2) were incubated for 48 h in routine conditions.

The cells of each group were collected, respectively, and then were labeled with Annexin-V-Fluos. After 15 min incubation in dark at room temperature, the cells' apoptosis was analyzed with the flow cytometer (FCM, Vantage SE, BD Company, USA).

#### 2.2.2. Cell Ultrastructure Examination

To detect cell ultrastructure changes, tumor tissues after treatment [1] were fixed in 4% glutaraldehyde, subsequently made into ultrathin sections, stained with uranyl acetate and lead citrate, and then viewed under a TEM (H600, Japan).

#### 2.2.3. Immunohistochemistry Examination

To further confirm the therapeutic effect of the combination regimen and explore the mechanisms by which the combining therapy inhibited tumor cell proliferation and induced cell apoptosis and the effect on tumor angiogenesis, survivin, Ki67, livin, bcl-2, p53, VEGF proteins, and MVD in tumor tissue with different interventions were investigated by immunohistochemistry (IHC) assay. The IHC procedures were followed as the histostain-plus kits. MVD in the tumor tissue after treatment was analyzed with the immunohistochemical “hot spots” method by labeling CD34 in the present study. In detail, the special antibody CD34 was employed to label endothelial cells by IHC, and then endothelial cells were counted under a light microscope (Japan, OLYMPUS). Finally, microvessels were indirectly measured [10, 11].

The immunohistochemistry results were evaluated as follows [12–14]. Livin, bcl-2, and VEGF proteins were expressed in cytomembrane or cytoplasm of the tumor cells. Survivin existed in cytomembrane, cytoplasm, or nuclei. P53 and Ki67 proteins were accumulated in nuclei. Light yellow to brown stain in the cytoplasm, cytomembrane, or nuclei was considered to be positive. No difference between the tumor and the ground was defined as the negative stain. Positive cells and total cells were counted, respectively, and then the positive cell percentage (PP) and positive index (PI) were calculated as the following formulas. PP = (positive cell number/the total number of tumor cells) × 100%.PI = SI × SC. Stained cells (SC) were scored according to PP (0 = no score; 11%–25% = 1; 26%–50% = 2; 51%–75% = 3; >75% = 4). Staining intensity (SI) was scored as follows: negative = 0; yellow = 1; claybank = 2; brown = 3. PI > 3 means it is positive.

#### 2.2.4. Histopathology Examination

Tumor tissue with different interventions was fixed in 4% neutral formaldehyde, then sectioned and stained with HE, and finally viewed with the light microscope for HE histopathological analysis.

#### 2.2.5. Statistical Analysis

Values are shown as mean ± SD. The data were analyzed with the SPSS 23.0 program. A *p* value of <0.05 was considered to be significant.

## 3. Results and Discussion

### 3.1. To Induce Tumor Cell Apoptosis

Inducing cancer cell apoptosis is one of the usual antitumor mechanisms for chemotherapy and radiotherapy, while the thermotherapy not only can induce cell apoptosis itself but also can obviously strengthen the cell apoptosis induced by radiotherapy and chemotherapy. The results of flow cytometry analysis showed that MFH, internal irradiation of nuclide 131I, and suicide gene TK driven by HRE/Egr1 all induced hepatoma cell apoptosis, but the joint application of these three took a much stronger effect. The cell apoptosis rate of the radionuclide-gene-MFH group reached 80.36%, significantly higher than 14.44% of the nuclide group, 23.13% of the MFH group, 25.83% of the radiation-gene group, and 5.68% of the negative control group (Table 1).

**Figure 1:**
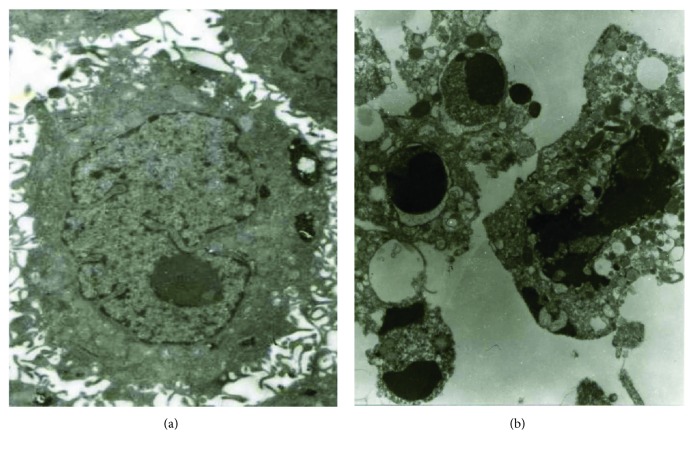
Ultrastructure of HepG2 cells (observed by TEM). (a) Cell without treatment (×15000). (b) Cell treated with radionuclide-gene-MFH for 48 h. Chromatin condensation, apoptotic bodies, and cytoplasmic vacuolation were observed (×10000).

Cell ultrastructure examination by TEM (H600, Japan) also certified representative morphological features of apoptotic cells including karyopyknosis, chromatin condensation, apoptotic body, and cytoplasmic vacuolation in the nuclide-gene-MFH group (Figure 1(b)). In contrast, hepatoma cells of the saline control group were regular in shape, with large nucleolus and fine chromatin (Figure 1(a)), which suggests inducing cancer cell apoptosis is also one of the antitumor mechanisms for the radionuclide-gene combined with MFH to treat hepatoma effectively, and the joint of radionuclide, suicide gene HSV-TK, and MFH can have an excellent synergistic effect on apoptotic inducing.

To further confirm the apoptotic induction of the combination therapy and explore how it induced tumor cell apoptosis with a synergy at the molecular level, we detected the expression of some proteins related to apoptosis such as survivin, bcl-2, livin, and p53 after treatment by IHC in this study.

As the strongest inhibitory factor of apoptosis ever discovered, survivin is closely related to cancer development and prognosis. It is scarcely expressed in normal tissue, but overexpressed in many malignant tumors such as hepatoma and gastric cancer. By inhibiting the activity of apoptotic protease, the overexpressed survivin protein can support tumor cells to escape from the checkpoint monitoring of cell cycle G2/M transition and resist cell apoptosis from DNA damage or mutation, resulting in abnormal cell division and proliferation [15–18]. Bcl-2, the antiapoptosis gene which is studied most widely and most deeply so far, is extensively expressed in many human malignant tumors. As a key regulator in apoptosis, bcl-2 can widely suppress various stimuli to inhibit cell apoptosis, thus getting cell life extended. Overexpressed bcl-2 can enhance cell resistance to most DNA damage, but it cannot prevent the damage itself and also cannot promote DNA damage repair. It has been confirmed that bcl-2 can restrain apoptosis mediated by p53 protein which is a molecular sensor of DNA damage but cannot inhibit p53 from moving to the nucleus and the growth arrest mediated by p53. Therefore, bcl-2 may play a role in keeping the signals of apoptosis activated from reaching target molecule after DNA damage [19–23]. P53 is a gene which has the closest correlation with the human tumor ever discovered so far. It is strongly related to apoptotic regulation. The wild type functioned as molecular police, monitoring the cell genome integrity. Once DNA is injured, p53 protein will accumulate and activate the repair system to repair the damaged DNA. If the repair fails, p53 protein will induce the cell apoptosis to death. Once missing or mutating, p53 gene will lose the monitoring function and cannot prevent the cell from proliferating or inducing apoptosis. As a result, the damaged cells with mismatched DNA enter the S phase, which results in gene mutation and chromosome aberration, and finally carcinogenesis appears. However, half-life of wild-type p53 protein is too short to be detected by IHC, while mutant p53 protein can be easily detected by IHC for its long half-life. Thus, p53 protein detected by IHC in cancer tissue is usually considered to be the mutant p53 gene product. Studies have shown that most cancers are accompanied with mutant p53 overexpressed. The excessive mutant p53 protein not only loses the role of “police” but also serves as a tumor-promoting factor to resist and eliminate the functions of normal p53 [24–26]. Livin, a kind of apoptosis-inhibiting factor newly found, can inhibit the caspase 3/7 and caspase 9 activity to block the apoptotic pathways based on the apoptotic receptor or mitochondria. It is barely expressed in human normal tissue but overexpressed in a number of human malignant tumors including hepatoma [12, 27, 28].

**Figure 2:**
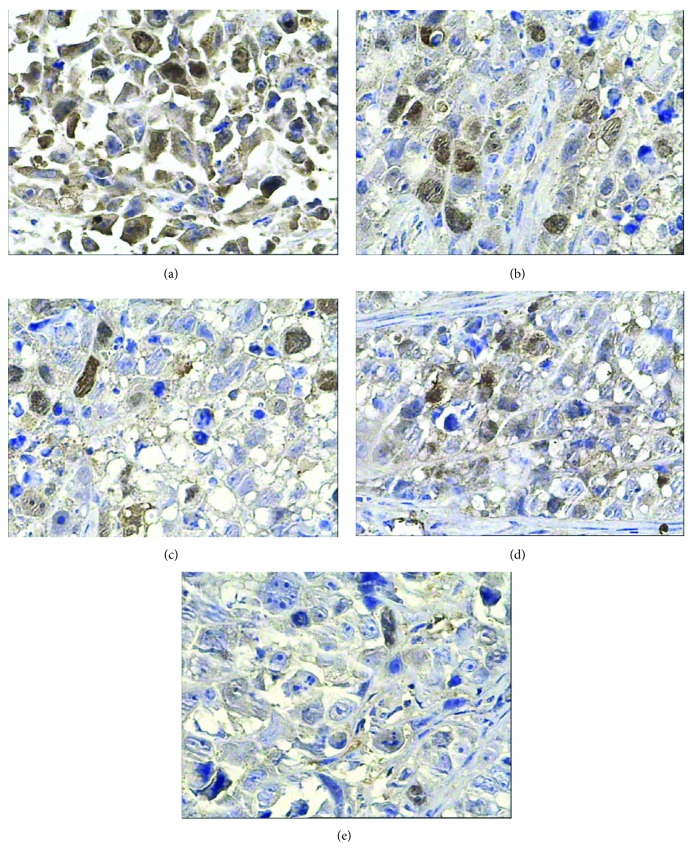
Survivin protein expression in hepatoma tissues after different treatments tested by IHC (400x). (a) Saline control group. (b) Radionuclide group. (c) Radionuclide-gene group. (d) MFH group. (e) Radionuclide-gene-MFH group.

**Figure 3:**
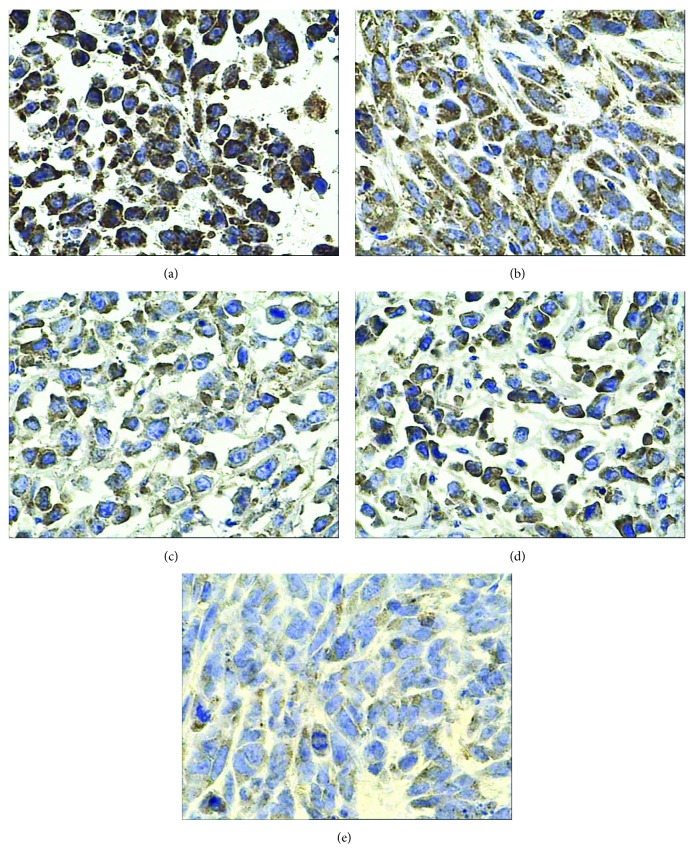
Livin protein expression in hepatoma tissues after different treatments tested by IHC (400x). (a) Saline control group. (b) Radionuclide group. (c) Radionuclide-gene group. (d) MFH group. (e) Radionuclide-gene-MFH group.

**Figure 4:**
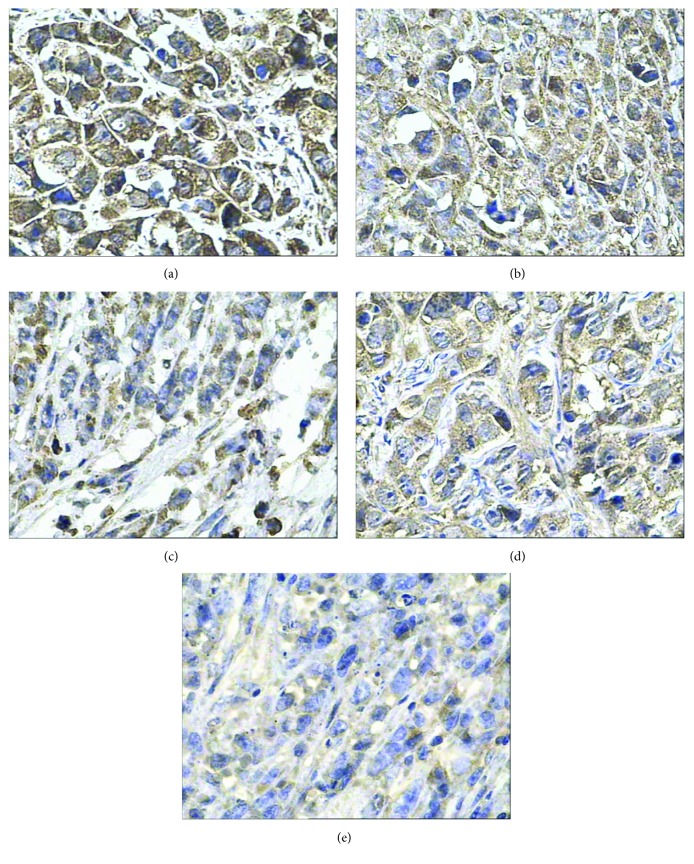
Bcl-2 protein expression in hepatoma tissues after different treatments tested by IHC (400x): (a) Saline control group. (b) Radionuclide group. (c) Radionuclide-gene group. (d) MFH group. (e) Radionuclide-gene-MFH group.

**Figure 5:**
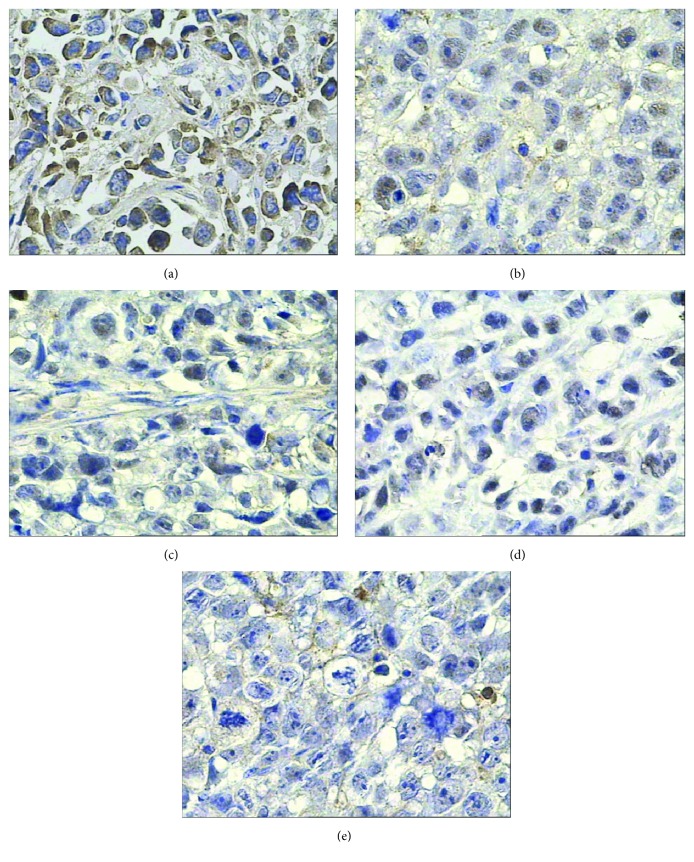
p53 protein expression in hepatoma tissues after different treatments tested by IHC (400x). (a) Saline control group. (b) Radionuclide group. (c) Radionuclide-gene group. (d) MFH group. (e) Radionuclide-gene-MFH group.

The results of IHC in this study showed that survivin, bcl-2, p53, and livin proteins were all positive in the saline control group. Bcl-2 and livin proteins stained with granular claybank or brown were in the cell membrane or cytoplasm, and survivin protein with granular claybank or brown staining was expressed in the cell membrane, cytoplasm, or nucleus, and p53 protein existed in the nucleus. Compared with the control group, survivin, bcl-2, livin, and p53 protein expression was weakened in different degrees in all the experimental groups, showing a smaller number of positive cells, lighter staining, and lower PI. PP of bcl-2 protein in the radionuclide group has no statistical difference from the saline control group (*p* > 0.05), but its positive intensity decreased obviously and its PI fell from 12 to 8. PP of survivin, bcl-2, livin, and p53 proteins in the other experimental groups was all statistically different from the saline control group (*p* < 0.05) except PP of bcl-2 protein in the radionuclide group, but the four proteins' expression in radionuclide-gene-MFH decreased most significantly, and their PI was only 1, which indicates these genes' expression has been completely inhibited (Figures 2–5 and Tables 2–5). This reveals that internal exposure of the radionuclide, radiation-gene therapy, and MFH inducing hepatoma apoptosis may be realized by downregulated expression of survivin, bcl-2, p53, and livin gene. The combination of three therapies can get the most lethal apoptosis, with an obvious synergistic effect.

### 3.2. To Inhibit Tumor Cell Proliferation

The most fundamental biological characteristic of malignant tumor is immortalization, and the proliferative activity is closely related to tumor biological behavior, particularly to tumor invasion, metastasis, and prognosis. Our previous findings, such as the inhibition rate of cell proliferation, the cell morphology changes, and the mass inhibition rate and volume inhibition rate of xenograft hepatoma, showed that both monotherapy and combination therapy had antiproliferative effects on hepatoma, but the targeted combination therapy of 131I, HSV-TK, and MFH using magnetic nanoparticles (Mn_0.5_Zn_0.5_Fe_2_O_4_) as carriers obtained the strongest inhibition effect, far better than any involved monotherapy [1].

**Figure 6:**
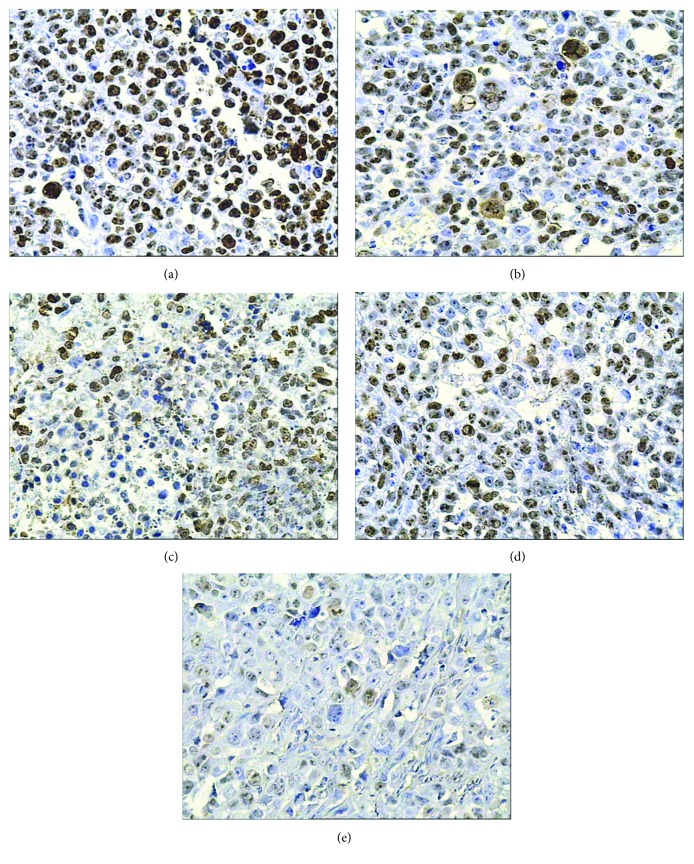
Ki67 protein expression in hepatoma tissues after different treatments tested by IHC (200x). (a) Saline control group. (b) Radionuclide group. (c) Radionuclide-gene group. (d) MFH group. (e) Radionuclide-gene-MFH group.

Ki-67 is a nuclear proliferation marker which is involved in mitosis. It presents an obvious periodism in cell cycle: no expression is in G0 phase, then appearing in G1 mid phase or late phase, and then gradual increasing in the S phase and the G2 phase, subsequently reaching the peak at the M phase, and finally rapidly disappearing in the late phase of the cell division. Because of its short half-life and rapid degradation after a cell cycle, Ki-67 protein is recognized as one of the most reliable indicators to evaluate tumor cells' proliferative activity. As a rule, the more Ki-67 is, the brisker the cells proliferate. Its label index has been applied to assess the efficacy of cancer treatment [29–31]. Thus, Ki67 protein is chosen to represent the nuclear proliferation level and tested in this study. The results showed that MFH, internal exposure of the radionuclide, radionuclide-gene therapy, and their combination all downgraded Ki67 expression, but Ki67 expressed least in the combined therapy group (the radionuclide-gene-MFH group) and its proliferation index was much smaller than that of any other groups (Figure 6 and Table 6). It is so inferred that downgrading Ki67 expression may be one of the ways to inhibit hepatoma proliferation. Its mechanism may be involved in the following. Lowering the expression of Ki67 means inhibiting DNA synthesis, thus restraining the replication of tumor cytoplasm and nuclei, eventually leading to cell mitosis blocking and cell proliferation inhibiting. The combination in terms of internal exposure of radionuclide, radionuclide-gene therapy, and MFH got the strongest inhibitory effect on Ki67 expression, with an obvious synergistic effect.

### 3.3. To Inhibit Tumor Angiogenesis

Tumor growth and metastasis depend on tumor neovascularization. In general, the tumor without angiogenesis mainly relies on the adjacent blood flow system to supply oxygen and nutrients by dispersion and carry away metabolites. Under this circumstance, the tumor cell apoptosis rate is usually extremely high and the tumor is dormant or eventually regressive. However, once new blood vessels produce, the tumor will rapidly grow and even metastasize. VEGF and MVD are both good indicators to reflect tumor angiogenesis [32–35].

VEGF, a specific mitogen for vascular endothelial cell, is one of the most important positive regulation factors in angiogenesis process, playing an important role in tumor angiogenesis. Its function is mainly involved in three aspects: (1) increasing vascular permeability. To be specific, VEGF is the strongest penetration enhancer ever known, and it can increase the permeability of blood vessels, facilitate plasma proteins and fibrinogen extravasation, cause extracellular matrix change, and finally promote new angiogenesis and new matrix formation; (2) promoting the mitosis of vascular endothelial cell and stimulating vascular endothelial cell proliferation and angiogenesis; and (3) VEGF can induce the expression of protein hydrolysis enzyme, tissue factors, and stromal collagen enzyme, activate the factor VII to release, change the extracellular matrix, and mediate endothelial cell migration and invasion. Thus, it is conducive to angiogenesis. In a word, VEGF from tumor cells not only can induce tumor angiogenesis but can also change the structure of new blood vessels and thus cause vessels' abnormal function so as to support the tumor growth and help tumor cells “escape” to metastasis. Studies have shown that VEGF expression is extremely low in normal tissue, while significantly high in hepatoma and other malignancies. VEGF protein has been applied as an indicator for tumor prognosis and metastasis [36, 37]. MVD is a widely used indicator to evaluate angiogenesis degree and reflect the potential ability of tumor infiltration, relapse, and metastasis in a certain extent. It can be used as an independent predictor for tumor prognosis [38, 39]. CD34 is the most popular and special marker of vascular endothelial cell, and its expression level can directly reflect MVD of tumor. As a rule, the more CD34 is, the higher MVD is. The immunohistochemical “hot spot” method is usually adopted for MVD analysis by CD34 protein examination [10, 11]. To explore the effect of MFH, radionuclide 131I, suicide gene HSV-TK, and their combination on hepatoma angiogenesis, VEGF protein is investigated by IHC, and MVD changes are analyzed by the immunohistochemical “hot spot” method by labeling CD34 in the tumor tissues after treatment in the present study.

**Figure 7:**
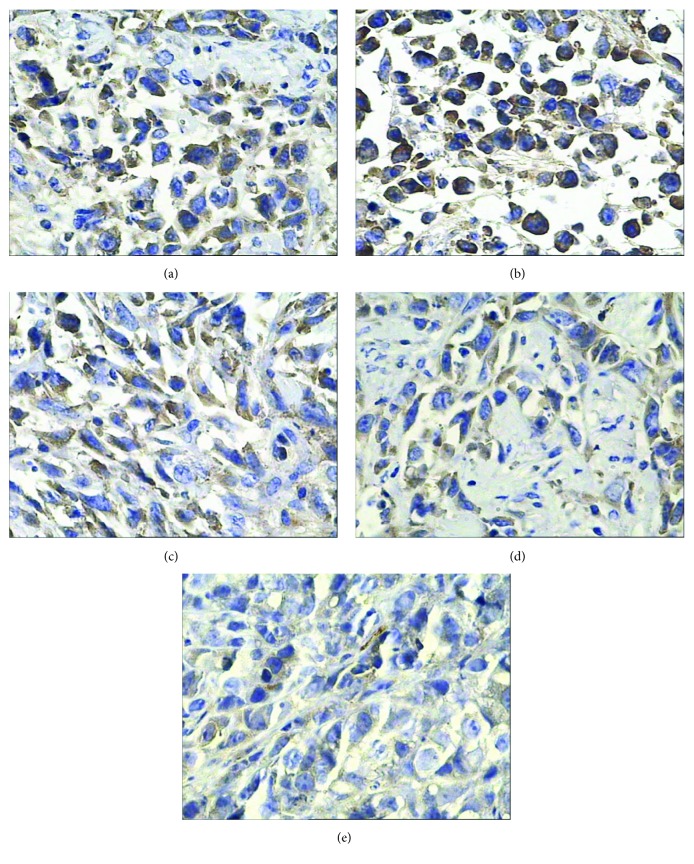
VEGF protein expression in hepatoma tissues after different treatments tested by IHC (400x). (a) Saline control group. (b) Radionuclide group. (c) Radionuclide-gene group. (d) MFH group. (e) Radionuclide-gene-MFH group.

**Figure 8:**
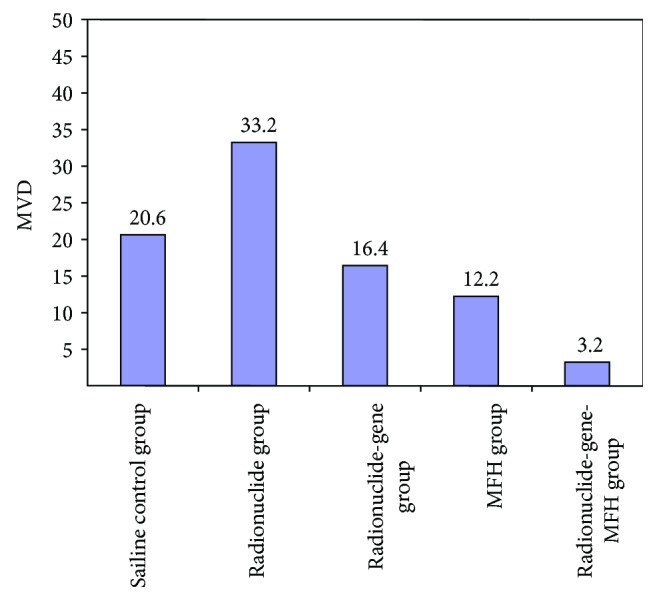
MVD in hepatoma tissues after different treatments.

**Figure 9:**
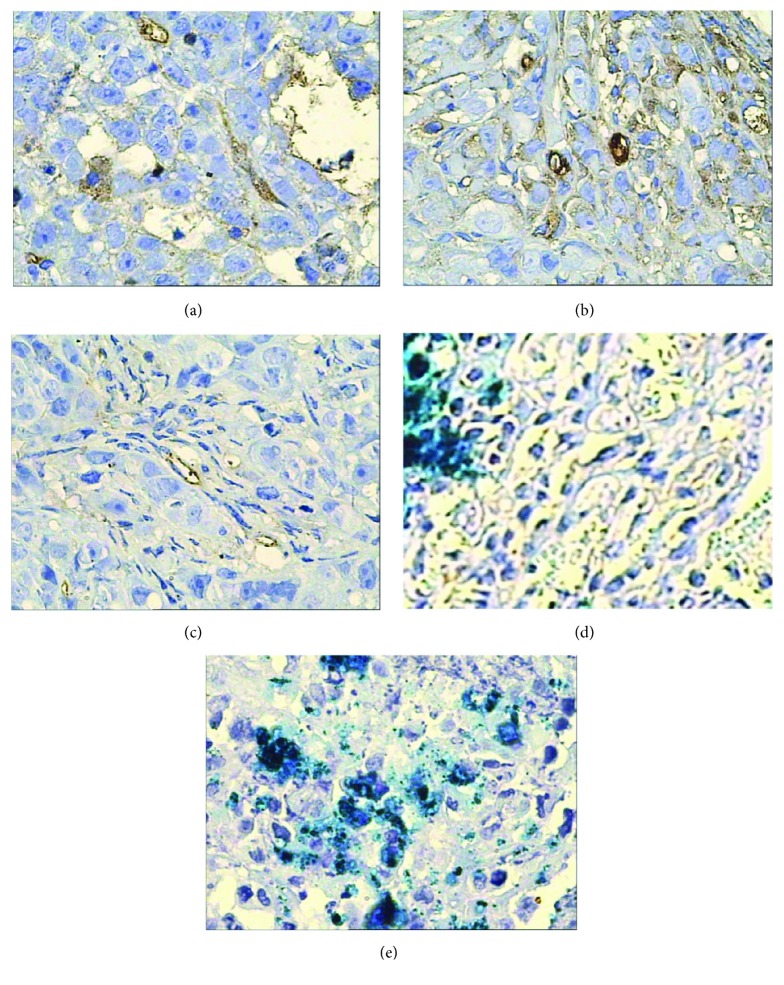
CD34 in hepatoma tissues after different treatments tested by IHC (400x). (a) Saline control group. (b) Radionuclide group. (c) Radionuclide-gene group. (d) MFH group. (e) Radionuclide-gene-MFH group.

**Figure 10:**
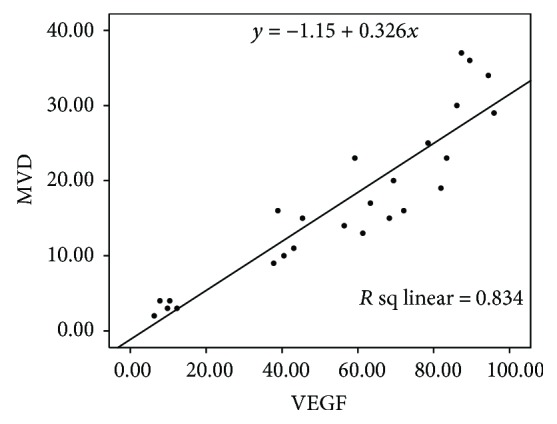
The correlation between VEGF and MVD in hepatoma tissues after different treatments.

As shown in Figure 7 and Table 7, PP and PI of VEGF protein were 90.64 ± 4.33% and 12 in the radionuclide group, respectively, significantly higher than that in the control group 77.06 ± 6.10% and 8. MVD of the radionuclide group was 33.2 ± 3.56%, significantly higher than that of the control group 20.6 ± 3.51% (Figure 8), which suggests that nuclide treatment increased VEGF gene expression and promoted micrangium formation. This may be the tumor's self-protection for survival to minimize the radiation toxicity on tumor blood vessels. It also may be the hypoxia adaptivity caused by radiation. Both VEGF protein and MVD in the other experimental groups decreased and their PP and PI of VEGF and MVD were all smaller than that in the radionuclide group and the control group (*p* < 0.05), but it is the smallest in the radionuclide-gene-MFH group. After radionuclide-gene therapy in combination with magnetic fluid hyperthermia, PP and PI of VEGF protein were only 9.32 ± 2.33% and 1, respectively, and MVD was merely 3.2 ± 0.84%, much lower than that in any other groups (*p* < 0.05) (Figures 7–9 and Table 7). Deep blue magnetic materials stained with Prussian blue were observed in the tumor tissue of the MFH group and the radionuclide-gene-MFH group (Figures 9(d) and 9(e)). Linear correlation analysis showed a significantly positive correlation between VEGF and MVD (Figure 10, *r* = 0.913), further confirming the important effect of VEGF on tumor angiogenesis. The above data suggest that the suicide gene and magnetic fluid hyperthermia both can downgrade VEGF expression, inhibit angiogenesis, lower microvascular density, and resist the increase of VEGF expression and microvascular density induced by radionuclide, showing an obvious radiosensitization effect. However, the combined application of radionuclide, gene, and MFH displayed the strongest inhibitory effect on tumor angiogenesis, much better than any monotherapy involved, which indicates angiogenesis inhibition may be another important mechanism of radiation-gene therapy combined with magnetic fluid hyperthermia to treat hepatoma.

### 3.4. To Induce Tumor Cell Necrosis

**Figure 11:**
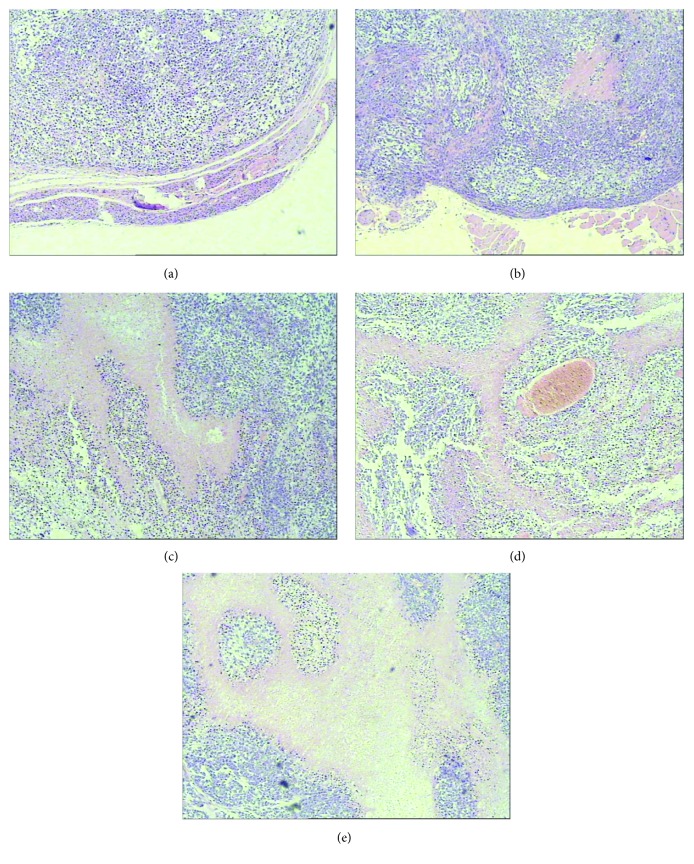
Xenograft hepatoma histopathological findings after different treatments (stained with HE, ×100). (a) Saline control group. (b) Radionuclide group. (c) MFH group. (d) Radionuclide-gene group. (e) Radionuclide-gene-MFH group.

The H&E staining assays showed that tumor cells in the saline control group were dense and actively proliferous. Hemorrhage and necrosis at different degrees were observed in all the experimental groups, but it is the most serious one in the radionuclide-gene-MFH group, with large area necrosis of cancer tissue like “map,” the necrosis area stained with red, coagulative necrosis of tumor cells, and disintegrative or disappeared nucleus. Many dotted or flake necrosis focuses were viewed in the radionuclide group, the MFH group, and the radionuclide-gene group (Figure 11).

Flow cytometry analysis demonstrated a certain degree of necrosis in each experimental group, and their necrosis rate was 5.89%, 9.99%, 9.35%, and 23.02% in the radionuclide group, the MFH group, the radionuclide-gene group, and the radionuclide-gene-MFH group, respectively. Obviously, the necrosis rate in the three combined group was the highest, significantly higher than that in any other groups, indicating nuclide, suicide gene, and magnetic induction heating all can induce hepatoma necrosis, but the three combined therapy may produce an obvious synergistic effect (Table 1). The mechanisms may be as follows. Hyperpyrexia may cause protein denaturation and damages of cell membrane, mitochondria membrane, lysosome membrane, and endoplasmic reticulum. Cell membrane damage may result in permeability increase, Na^+^ and Ca^++^ influx, and eventual cell swelling and necrosis. In turn, the precursor drug GCV, suicide gene HSV-TK, and radionuclide 131I easily enter cells and play the therapy role. Lysosome membranolysis may cause lots of acid lytic enzymes being released, leading to the further breaking of the cell membrane and cytoplasm outflux. Eventually, cells die. It is more important for mitochondrial damage. As chondriosomes in normal cells are usually less than that in tumor cells, once the chondriosome structure was damaged, aerobic metabolism of the tumor cell is disordered. Subsequently, ATP synthesis decreases and ATP enzyme dysfunctions, which eventually results in tumor cell degeneration or necrosis, while suicide gene HSV-TK and radionuclide 131I significantly intensified this role. In addition to angiogenesis inhibition of hyperpyrexia and suicide genes, based on anatomy characteristics, tumor blood vessels were sensitive to heat, radiation, and many chemotherapy drugs. Radiation-gene therapy in combination with magnetic fluid hyperthermia may also directly destroy the structure and the function of the tumor original vascular, leading to vasodilation, tissue congestion, blood flow stagnation, and eventual tumor tissue necrosis because of ischemia.

## 4. Conclusion

The combined hepatoma-targeted therapy of radionuclide, suicide gene, and MFH linked organically by PEI-MZF-NPs presented an obvious complementary synergy. It had a good anticancer effect, far better than any involved monotherapy. Its mechanism may be involved in the downregulation of Ki67 expression leading to tumor cell proliferation repression and inhibition of survivin, bcl-2, p53, and livin protein expression inducing tumor cell apoptosis, negatively regulating VEGF protein expression, and reducing vascular endothelial cells resulting in tumor angiogenesis inhibition and microvascular density decreasing and inducing tumor cell necrosis. These findings offer a basic data support and theoretical foundation for the clinical application of the combination therapy.

## Figures and Tables

**Table 1:** Flow cytometric analysis of apoptosis and necrosis in different therapy groups (%).

Group	Apoptosis rate	Necrosis rate
Blank control group	5.68	3.03
Radionuclide group	14.44	5.89
MFH group	23.13	9.99
Radionuclide-gene group	25.83	9.35
Radionuclide-gene-MFH group	80.36	23.02

**Table 2:** Survivin protein expression of hepatoma tissues after different treatments tested by IHC (mean ± s, *n* = 5).

Group	Saline group	Radionuclide group	Radionuclide-gene group	MFH group	Radionuclide-gene-MFH group
Positive percent (%)	90.36 ± 3.53^bcde^	58.90 ± 3.50^acde^	40.26 ± 3.60^abde^	49.48 ± 4.09^abce^	6.72 ± 1.54^abcd^
Positive index	12	9	4	4	1

^a^ *p* < 0.05 versus saline group; ^b^*p* < 0.05 versus radionuclide group; ^c^*p* < 0.05 versus radionuclide-gene group; ^d^*p* < 0.05 versus MFH group; ^e^*p* < 0.05 versus radionuclide-gene-MFH group.

**Table 3:** Livin protein expression of hepatoma tissues after different treatments tested by IHC (mean ± s, *n* = 5).

Group	Saline group	Radionuclide group	Radionuclide-gene group	MFH group	Radionuclide-gene-MFH group
Positive percent (%)	91.6 ± 4.21^cde^	86.90 ± 2.05^ce^	74.56 ± 2.06^abe^	79.98 ± 2.07^ae^	10.90 ± 1.75^abcd^
Positive index	12	8	3	8	1

^a^ *p* < 0.05 versus saline group; ^b^*p* < 0.05 versus radionuclide group; ^c^*p* < 0.05 versus radionuclide-gene group; ^d^*p* < 0.05 versus MFH group; ^e^*p* < 0.05 versus radionuclide-gene-MFH group.

**Table 4:** Bcl-2 protein expression of hepatoma tissues after different treatments tested by IHC (mean ± s, *n* = 5).

Group	Saline group	Radionuclide group	Radionuclide-gene group	MFH group	Radionuclide-gene-MFH group
Positive percent (%)	95.72 ± 2.07^cde^	92.30 ± 2.53^cde^	69.76 ± 3.11^abde^	84.46 ± 1.71^abce^	10.70 ± 2.19^abcd^
Positive index	12	8	3	4	1

^a^ *p* < 0.05 versus saline group; ^b^*p* < 0.05 versus radionuclide group; ^c^*p* < 0.05 versus radionuclide-gene group; ^d^*p* < 0.05 versus MFH group; ^e^*p* < 0.05 versus radionuclide-gene-MFH group.

**Table 5:** p53 protein expression of hepatoma tissues after different treatments tested by IHC (mean ± s, *n* = 5).

Group	Saline group	Radionuclide group	Radionuclide-gene group	MFH group	Radionuclide-gene-MFH group
Positive percent (%)	68.88 ± 3.59^bcde^	44.68 ± 2.29^acde^	22.10 ± 1.81^abe^	24.78 ± 1.80^abe^	3.58 ± 0.87^abcd^
Positive index	9	2	1	2	1

^a^ *p* < 0.05 versus saline group; ^b^*p* < 0.05 versus radionuclide group; ^c^*p* < 0.05 versus radionuclide-gene group; ^d^*p* < 0.05 versus MFH group; ^e^*p* < 0.05 versus radionuclide-gene-MFH group.

**Table 6:** Ki67 protein expression of hepatoma tissues after different treatments tested by IHC (mean ± s, *n* = 5).

Group	Saline group	Radionuclide group	Radionuclide-gene group	MFH group	Radionuclide-gene-MFH group
Positive percent (%)	79.02 ± 3.58^bcde^	42.44 ± 2.21^acde^	23.20 ± 2.53^abde^	29.38 ± 2.66^abce^	10.18 ± 1.64^abcd^
Positive index	12	8	3	4	1

^a^ *p* < 0.05 versus saline group; ^b^*p* < 0.05 versus radionuclide group; ^c^*p* < 0.05 versus radionuclide-gene group; ^d^*p* < 0.05 versus MFH group; ^e^*p* < 0.05 versus radionuclide-gene-MFH group.

**Table 7:** VEGF protein expression of hepatoma tissues after different treatments tested by IHC (mean ± s, *n* = 5).

Group	Saline group	Radionuclide group	Radionuclide-gene group	MFH group	Radionuclide-gene-MFH group
Positive percent (%)	77.06 ± 6.10^bcde^	90.64 ± 4.33^acde^	61.7 ± 4.49^abec^	41.14 ± 3.11^abce^	9.32 ± 2.33^abcd^
Positive index	8	12	6	4	1

^a^ *p* < 0.05 versus saline group; ^b^*p* < 0.05 versus radionuclide group; ^c^*p* < 0.05 versus radionuclide-gene group; ^d^*p* < 0.05 versus MFH group; ^e^*p* < 0.05 versus radionuclide-gene-MFH group.
